# Supplementary material for: PUREE: accurate pan-cancer tumor purity estimation from gene expression data
Source: Commun Biol. 2023 Apr 11;6:394. doi: 10.1038/s42003-023-04764-8 (PMC10090153; doi:10.1038/s42003-023-04764-8)
Supplement: Supplementary file 2 — Supplementary Information [file 42003_2023_4764_MOESM2_ESM.pdf]

Supplementary Information

Supplementary Note 1: Overview of existing transcriptomics-based approaches capable of predicting tumor purity

Purity prediction from gene expression matrix-like data is an example of a well-studied task of *computational deconvolution*[1]. Over the last few years, an abundance of techniques using bulk gene expression data as an input has been developed. However, only some of the existing methods are capable of performing deconvolution based solely on the input data matrix and a set of constraints included in the algorithm or in the reference data. Below is a brief overview of some widely used transcriptomics-based methods that are capable of predicting cancer cell proportions, given only gene expression matrix and, sometimes, other data included with the software.

CIBSERSORTx[2] works by first building a signature matrix of the most significant genes in pre-specified cell types and then, assuming a linear mixing model, performing ν-support vector regression to infer the proportions of cell types in each sample. Its most prominent limitation is that CIBSERSORTx relies on the signature matrix created from single-cell data and thus introduces certain bias into the deconvolution of samples from other experiments. Although the authors provide the signature matrix for some cancer types, for most of the new data the matrix has to be reconstructed de novo (however, the authors provide tools that might help with it).

EPIC[3] models the gene expression of the tumor tissue as the sum of the gene expression profiles from the pure cell types composing the sample and uses a constrained least square optimization to perform the deconvolution.

ESTIMATE[4] first pre-determines genes that are likely to define the unique expression patterns of the stromal cells (stromal signature) and the infiltrating immune cells (immune signature). It then uses single-sample Gene Set Enrichment Analysis (ssGSEA)[5] to calculate enrichment scores that serve as a proxy for the presence of infiltrating stromal and immune cells in the tumor samples. Finally, the calculated scores and purity values from a DNA-based method ABSOLUTE[6] are used to train a nonlinear least-squares method to derive the final formula for calculating the tumor purity. The potential downside of this approach is that the genes that are derived for the immune and stromal signature might not capture all of the necessary information required to predict malignant cell proportions. Additionally, ESTIMATE is trained on the estimates of ABSOLUTE, which was one of the earlier methods and might not provide the most accurate genomic-based estimates.

DeMixT[7] models the observed gene expression signal Y_{ig} as a sum of its constituent components:

$$Y_{ig} = \pi_{1,i}N_{1,ig} + \pi_{2,i}N_{2,ig} + \left(1 - \pi_{1,i} - \pi_{2,i}\right)T_{ig} \quad \text{(Eq. 1)}$$

for each gene g and each sample i. $\pi_{1,i}$ and $\pi_{2,i}$ are cell proportions, N_1, N_2 and T are gene expression values within respective cell types. N_1- and N_2-components are estimated from the available reference samples (normal samples provided as an input), while T_{ig} is derived via inference over a probabilistic model. However, the method's prior probabilistic assumptions are still approximations of the underlying biology, which might introduce additional bias.

LinSeed[8] constructs a collinearity network of genes to determine significantly mutually linear features and keep only them for a later simplex-based deconvolution approach. However, if cell types are closely related to each other, the simplex approach might have trouble distinguishing them as distinct corners (they would be simply too close in the sample vector space). Moreover,

our tests have shown that LinSeed's deconvolved cell type proportions are not constrained to sum-to-one, and can sometimes exceed reasonable values (be > 1 or < 0).

DeconRNASeq[9] uses quadratic programming to solve the weighted non-negative least squares problem $X = AS$ for the cell type proportion matrix A, where X is the input gene expression matrix and S is a cell type-specific expression signature matrix. Similar to the CIBERSORTx, the method's performance highly depends on the quality of the signature matrix provided.

In short, while the existing methods employ novel techniques and often use valid assumptions about the deconvolution process, there still are several potential areas for improvement, namely their low correlations with DNA-based estimates, potential bias in the feature selection approach, or being overly- or under-specialized for different cancer types.

Supplementary Note 2: Transcriptomics-based approaches for tumor purity prediction excluded from the study

There were other methods that we found highly relevant to our work but were not able to include in the benchmark.

Gbm.ensemble is a similar supevised method by Li et al.[10] built as an ensemble of XGBoost models trained on ABSOLUTE tumor purities. Unfortunately, we were not able to run the method following the instructions provided by the authors (https://github.com/yuanyuanli66/gbm.ensemble).

Koo and Rhee[11] built several machine learning models using consensus purity estimates from the literature (CPE values from the "Systematic pan-cancer analysis of tumour purity" by Aran et al.[12]). However, the Github page of their work (https://github.com/BonilKoo/ML_purity) does not present a working method and so this approach could not be added to the benchmark.

DeClust by Wang et al.[13] is a method for reference profile-free deconvolution method to infer cancer cell-intrinsic subtypes, that could potentially be used to estimate cancer cell proportion in the tissues. However, we were not able to run the method for the tumor purity estimation purposes using the code provided by the authors (https://github.com/integrativenetworkbiology/DeClust).

Abbreviation	Full name	Number of samples (in train set / in test set)	Median tumor purity (in train set / in test set)
BRCA	Breast invasive carcinoma	1060 (848 / 212)	0.56 (0.56 / 0.55)
LUAD	Lung adenocarcinoma	508 (406 / 102)	0.42 (0.42 / 0.44)
LGG	Brain lower grade glioma	506 (405 / 101)	0.66 (0.67 / 0.62)
HNSC	Head and neck squamous cell carcinoma	494 (395 / 99)	0.47 (0.47 / 0.47)
PRAD	Prostate adenocarcinoma	489 (391 / 98)	0.48 (0.48 / 0.5)
THCA	Thyroid carcinoma	485 (388 / 97)	0.52 (0.52 / 0.54)
LUSC	Lung squamous cell carcinoma	482 (385 / 97)	0.49 (0.49 / 0.48)
STAD	Stomach adenocarcinoma	410 (328 / 82)	0.47 (0.46 / 0.47)
BLCA	Bladder urothelial carcinoma	403 (322 / 81)	0.56 (0.57 / 0.56)
KIRC	Kidney renal clear cell carcinoma	377 (302 / 75)	0.51 (0.51 / 0.53)
CRC	Combined: colon adenocarcinoma (COAD) and rectum adenocarcinoma (READ)	367 (294 / 73)	0.62 (0.62 / 0.62)
SKCM	Skin cutaneous melanoma	365 (292 / 73)	0.64 (0.66 / 0.57)
LIHC	Liver hepatocellular carcinoma	360 (288 / 72)	0.66 (0.66 / 0.67)
OV	Ovarian serous cystadenocarcinoma	301 (241 / 60)	0.72 (0.72 / 0.76)
CESC	Cervical squamous cell carcinoma and endocervical adenocarcinoma	292 (234 / 58)	0.61 (0.6 / 0.63)
KIRP	Kidney renal papillary cell carcinoma	284 (227 / 57)	0.66 (0.66 / 0.61)
ESCA	Esophageal carcinoma	180 (144 / 36)	0.56 (0.54 / 0.59)
UCEC	Uterine corpus endometrial carcinoma	179 (143 / 36)	0.67 (0.66 / 0.67)
PAAD	Pancreatic adenocarcinoma	176 (141 / 35)	0.35 (0.36 / 0.35)
GBM	Glioblastoma multiforme	146 (117 / 29)	0.72 (0.73 / 0.72)

Suppl. Table 1: TCGA dataset description and train/test set distribution. TCGA dataset used in this study was compiled from datasets of 20 solid cancer types, resulting in 7864 samples (6291 in the train set, 1573 in the test set). Median purity is rounded to 2 digits after the decimal point.

Cohort name	Cancer type	Num. of samples	Median purity	Purity estimation method
TCGA (test set)	20 solid cancer types	1573	0.56	Consensus (ABSOLUTE, ASCAT, PurBayes, AbsCNSeq)
Chen et al.	Lung	172	0.45	Sequenza
Chua et al.	Lung	64	0.6	Consensus (theta2, TitanCNA, PurBayes, AbsCNSeq)
Joanito et al.	Colorectal	153	0.62	Consensus (theta2, TitanCNA, PurBayes, AbsCNSeq)
TCGA-CRC+	Colorectal	243	0.7	ABSOLUTE
TCGA-UCEC+	Uterine	353	0.76	ABSOLUTE
TCGA-PCPG	Paraganglioma	164	0.74	ABSOLUTE
TCGA-TGCT	Testicular	155	0.6	ABSOLUTE

Suppl. Table 2: Independent datasets descriptions and purity statistics. Median purity is rounded to 2 digits after the decimal point.

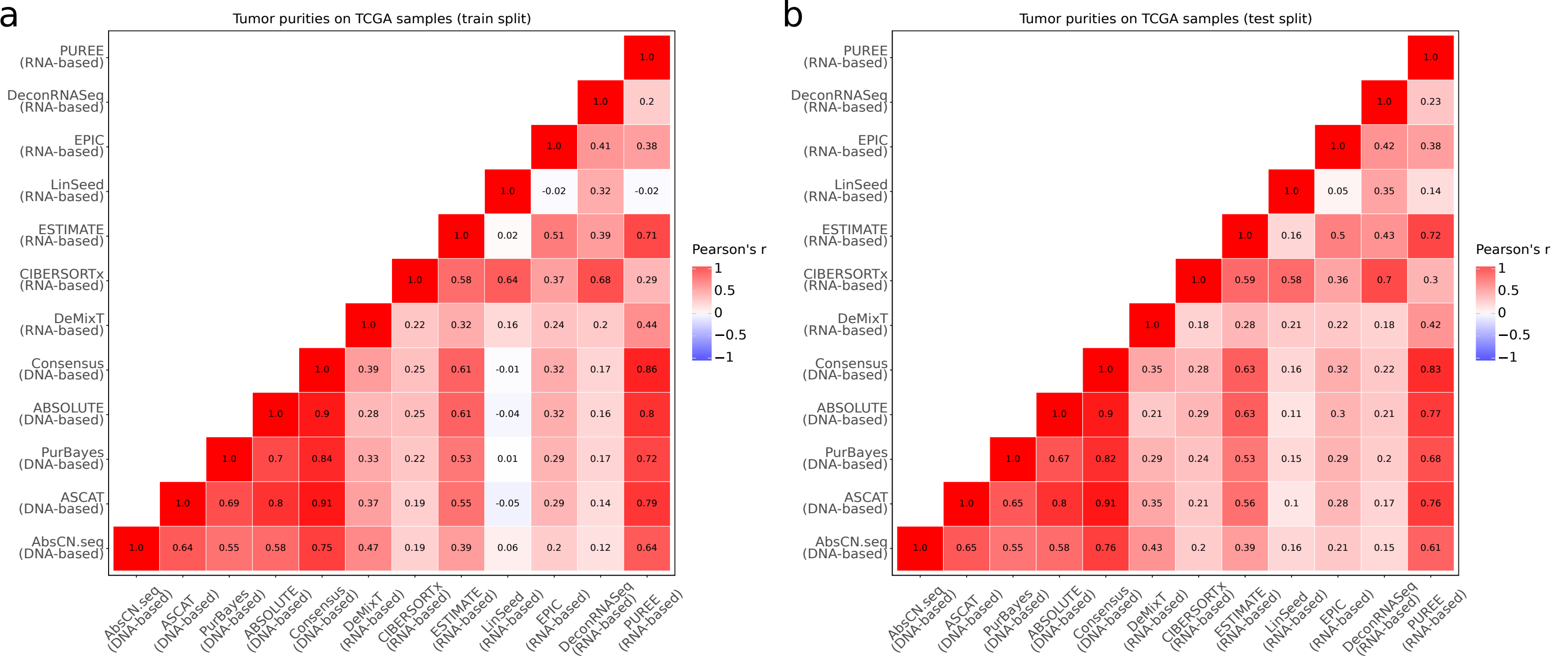

Suppl. Figure 1: Agreement of the genomics- and transcriptomics-based purity estimation methods. Pairwise correlations of tumor purity estimates were computed separately for **a)** TCGA train (6291 samples) and **b)** TCGA test (1573 samples) splits of the dataset to account for the machine learning training procedure of PUREE.

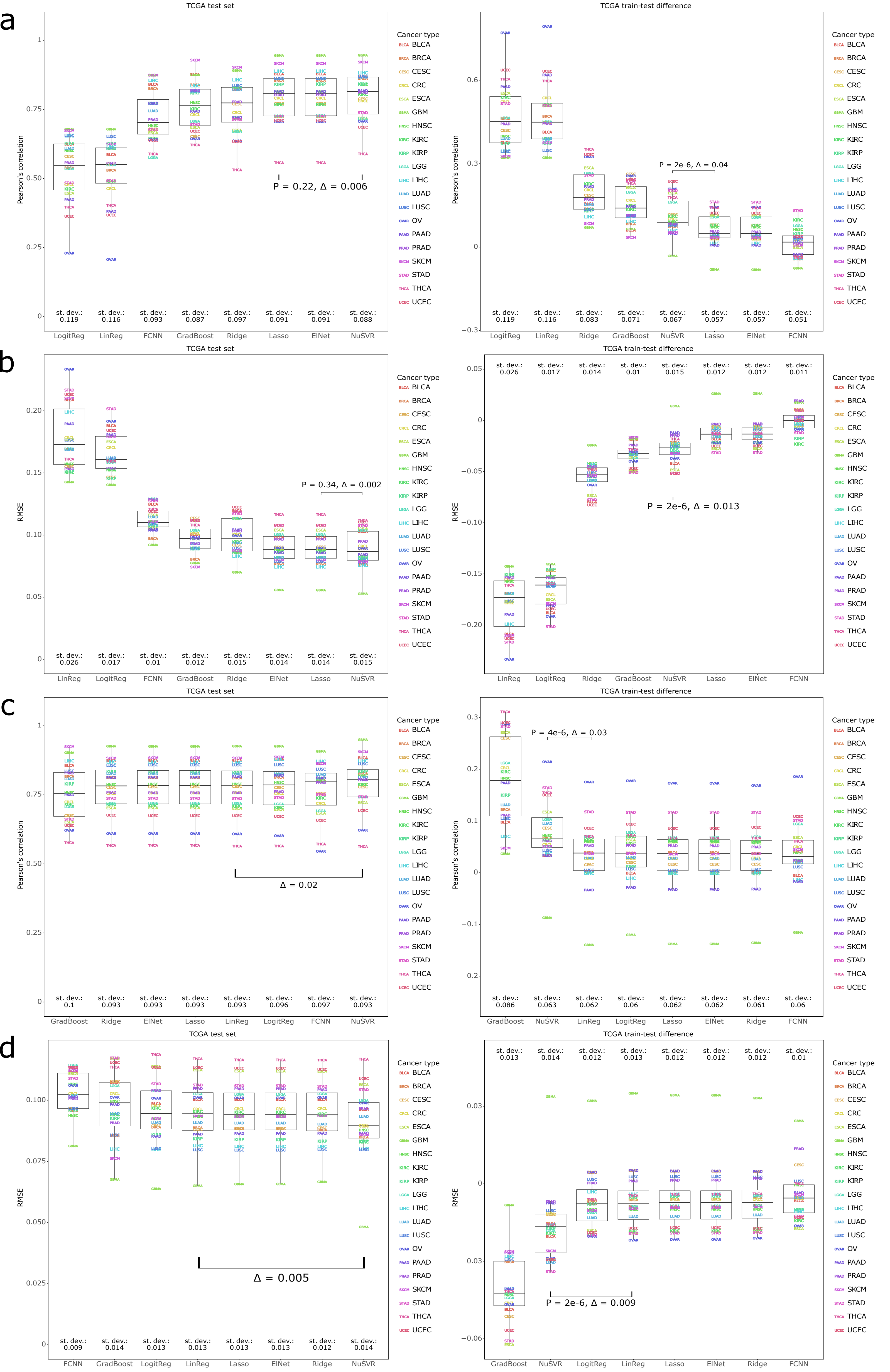

Suppl. Figure 2: Exploring the range of machine learning methods for the PUREE approach. a,b) models trained on the full set of 9554 features (a - Pearson's correlation, b - RMSE); lasso was chosen as the best for the full feature set due to good performance and low train-test difference in performance. **c,d)** models trained on the reduced feature set of 158 features (c - Pearson's correlation, d - RMSE); linear regression was chosen over NuSVR due to comparable performance, lower train-test difference in performance and simplicity. Each column depicts performance on the TCGA test set and the difference in performance between train and test sets respectively. Gradient Boosting, NuSVR, Ridge, Lasso and Elastic Net models were trained using 5-fold cross-validation hyperparameter selection procedure. In the boxplots, the lower and upper hinges correspond to the first and third quartiles, the upper whisker extends to the largest value no further than 1.5 of inter-quartile range from the hinge, the lower whisker extends to the smallest value no further than 1.5 of inter-quartile range from the hinge, and points beyond the end of the whiskers are plotted individually. Numbers next to boxplots indicate standard deviation. Delta between boxplots medians and Wilcoxon signed-rank test (two-tailed) shown for two best models in each plot (NuSVR and Lasso for 10K and NuSVR and Linear Regression for the reduced set).

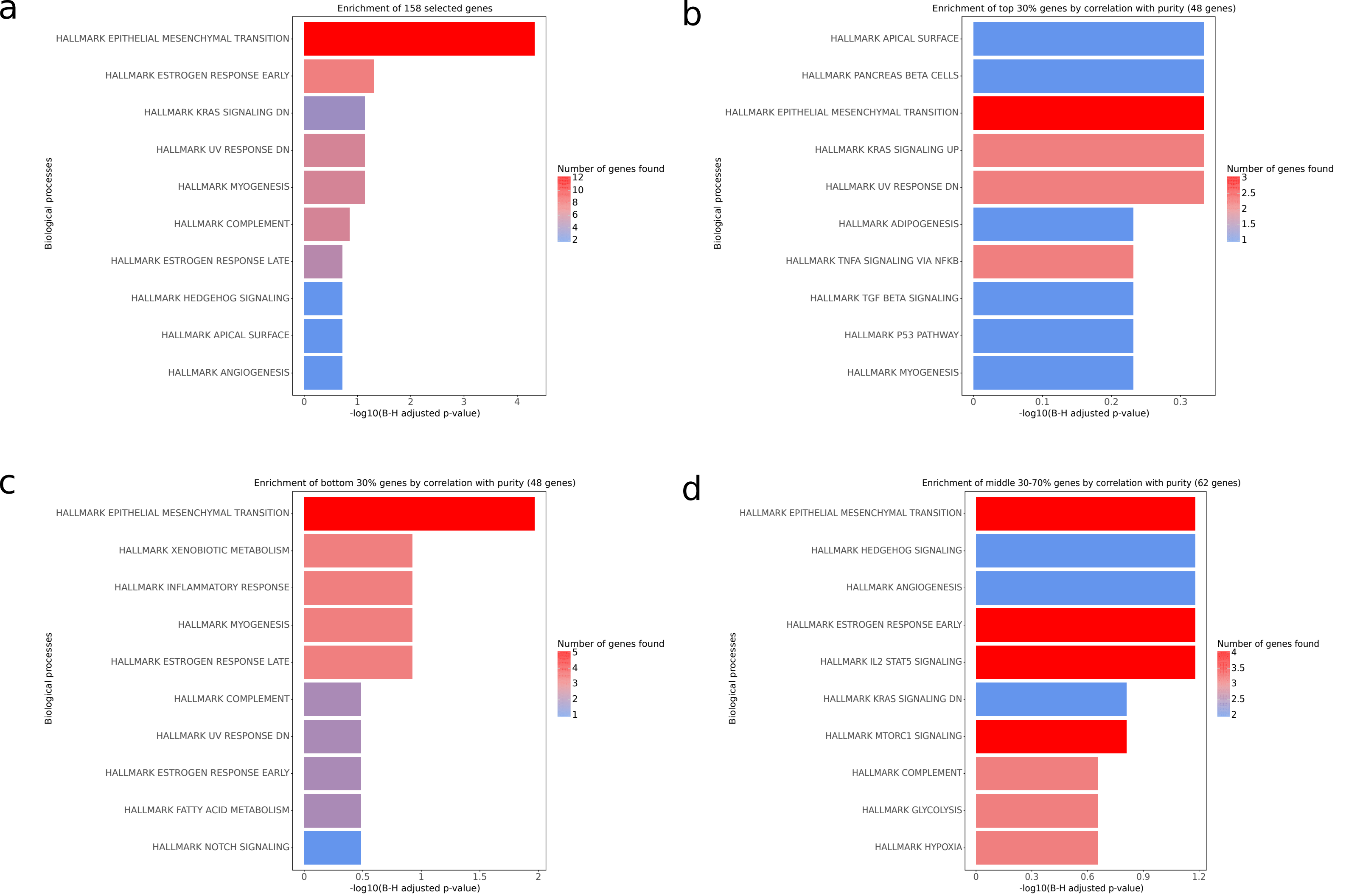

Suppl. Figure 3: Hallmark pathway analysis of 158 **genes used in the final model.** Only the top 10 pathways ranked by Benjamini-Hochberg adjusted P-value shown. **a)** enrichment analysis of 158 selected genes; **b)** enrichment analysis of top 30% genes by expression-purity correlations (correlation above 0.05); **c)** enrichment analysis of bottom 30% genes by expression-purity correlations (correlation below -0.14); **d)** enrichment of genes whose expression-purity correlations lie in the 30-70% ranking interval (correlation between 0.05 and -0.14). Enrichment analysis performed with GSEApy package in Python. Expression-purity correlations calculated using averages of mean correlations per cancer type. Pearson's correlation used.

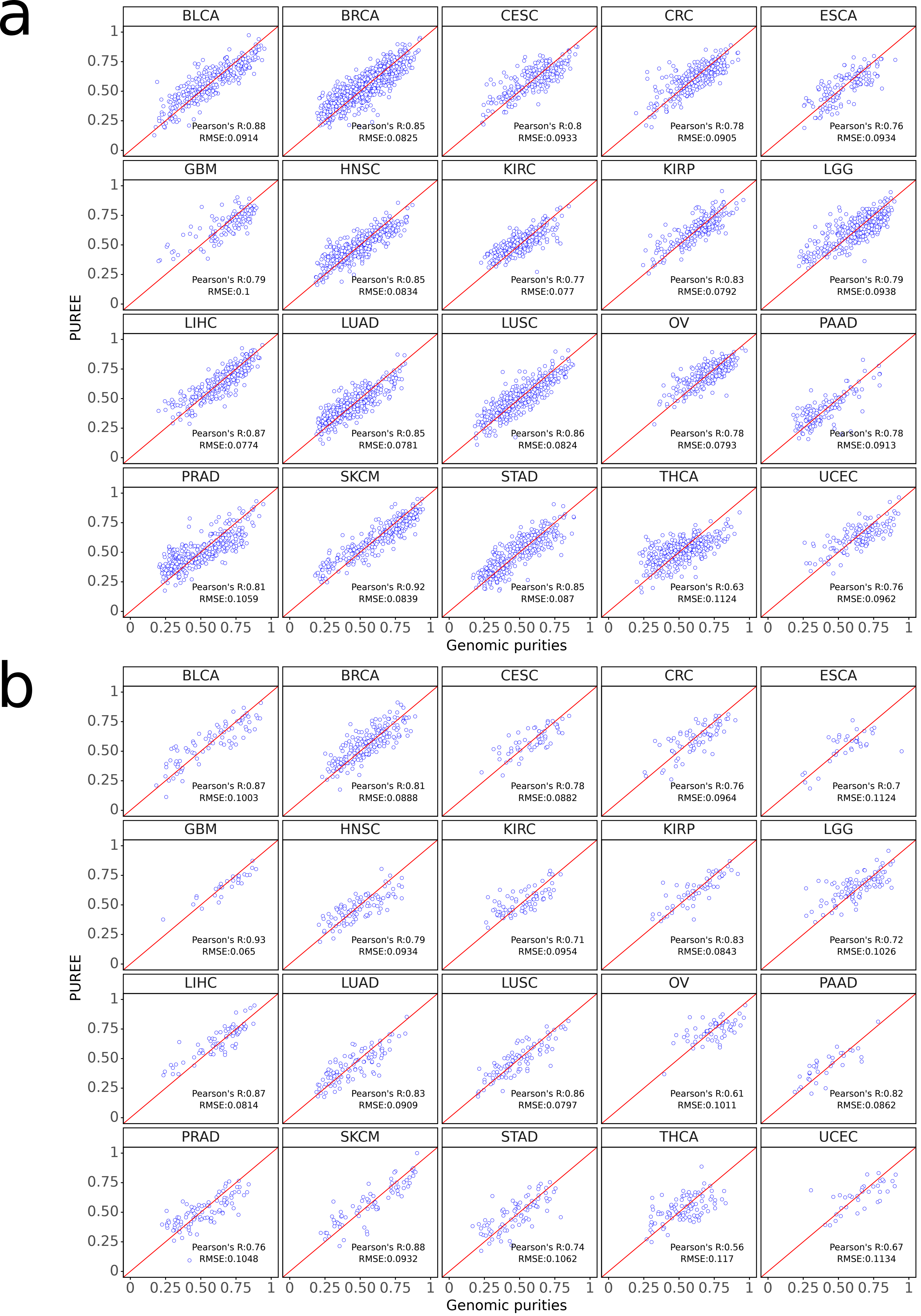

Suppl. Figure 4: Raw data of purity predictions of PUREE across 20 cancer types from TCGA. a) TCGA train split (6291 samples). **b)** TCGA test split (1573 samples). The red diagonal lines are drawn from (0,0) to (1,1), indicating the ideal prediction case.

a

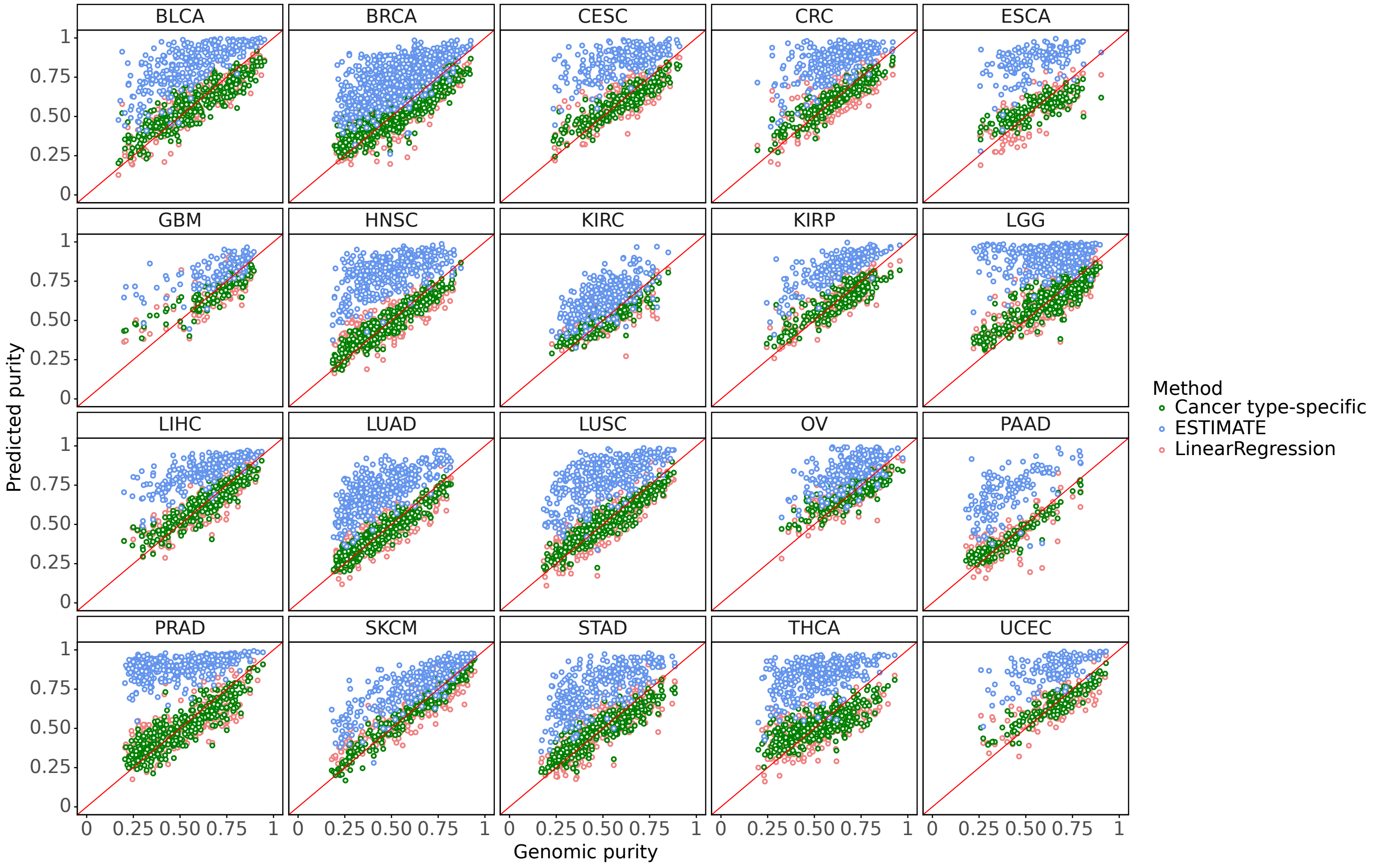

b

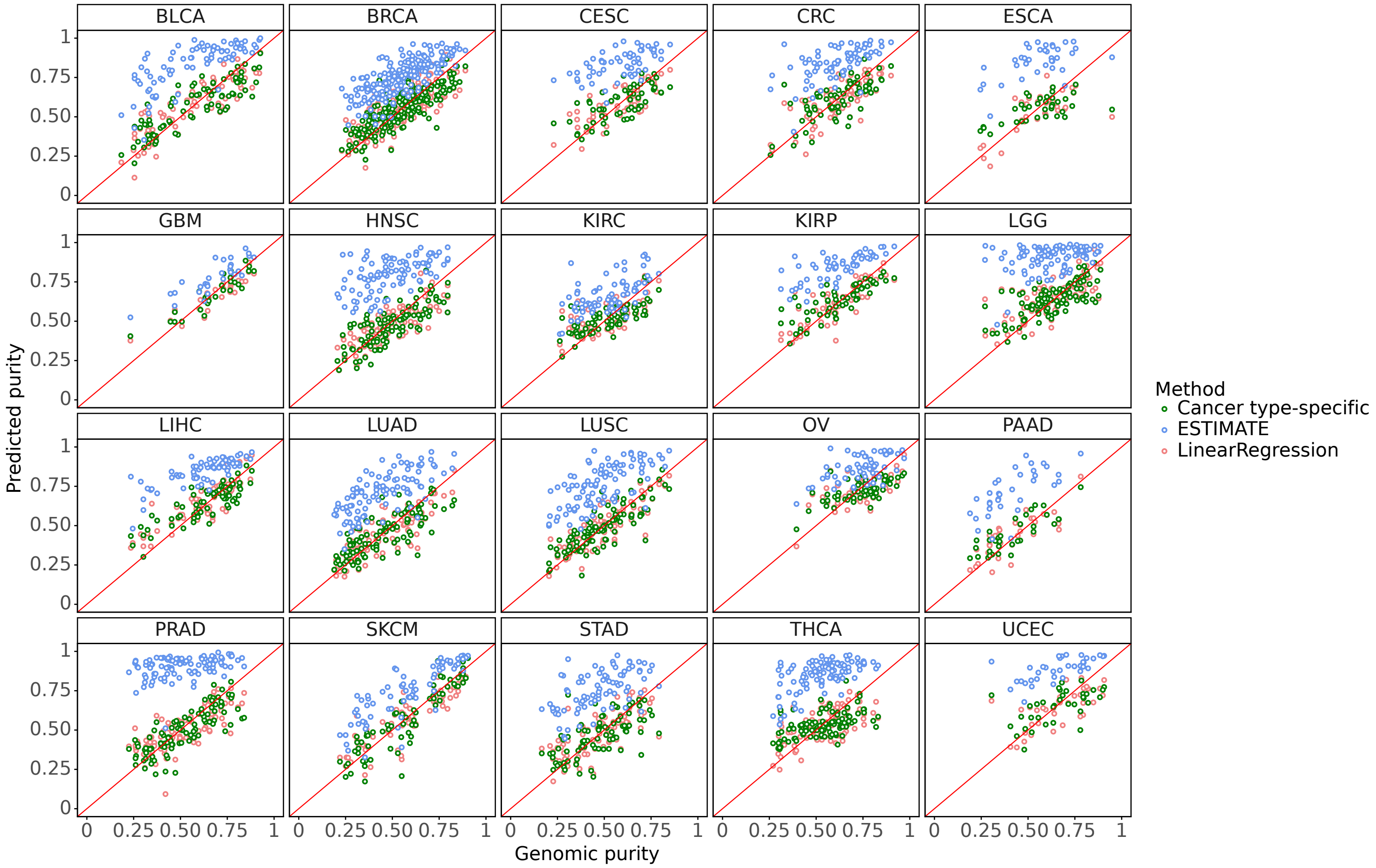

Suppl. Figure 5: Raw data of purity predictions of cancer-specific models, PUREE and ESTIMATE across 20 cancer types from TCGA on **a)** TCGA train set (6291 samples) and **b)** TCGA test set (1573 samples). The red diagonal lines are drawn from (0,0) to (1,1), indicating the ideal prediction case.

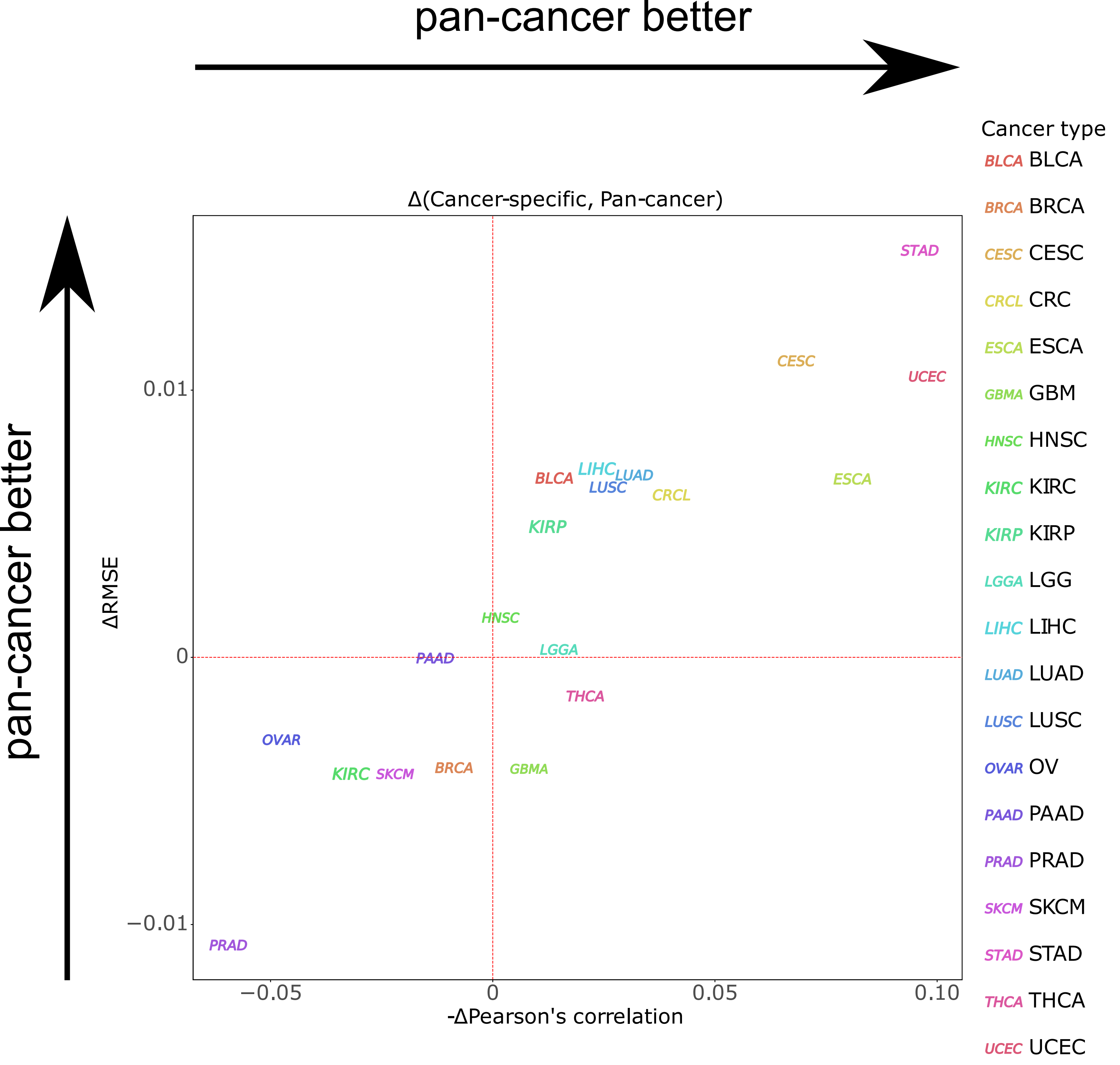

Suppl. Figure 6: Cancer type-specific vs pan-cancer test. Delta between average metrics per cancer type between the pancancer model (PUREE) and the cancer type-specific ones.

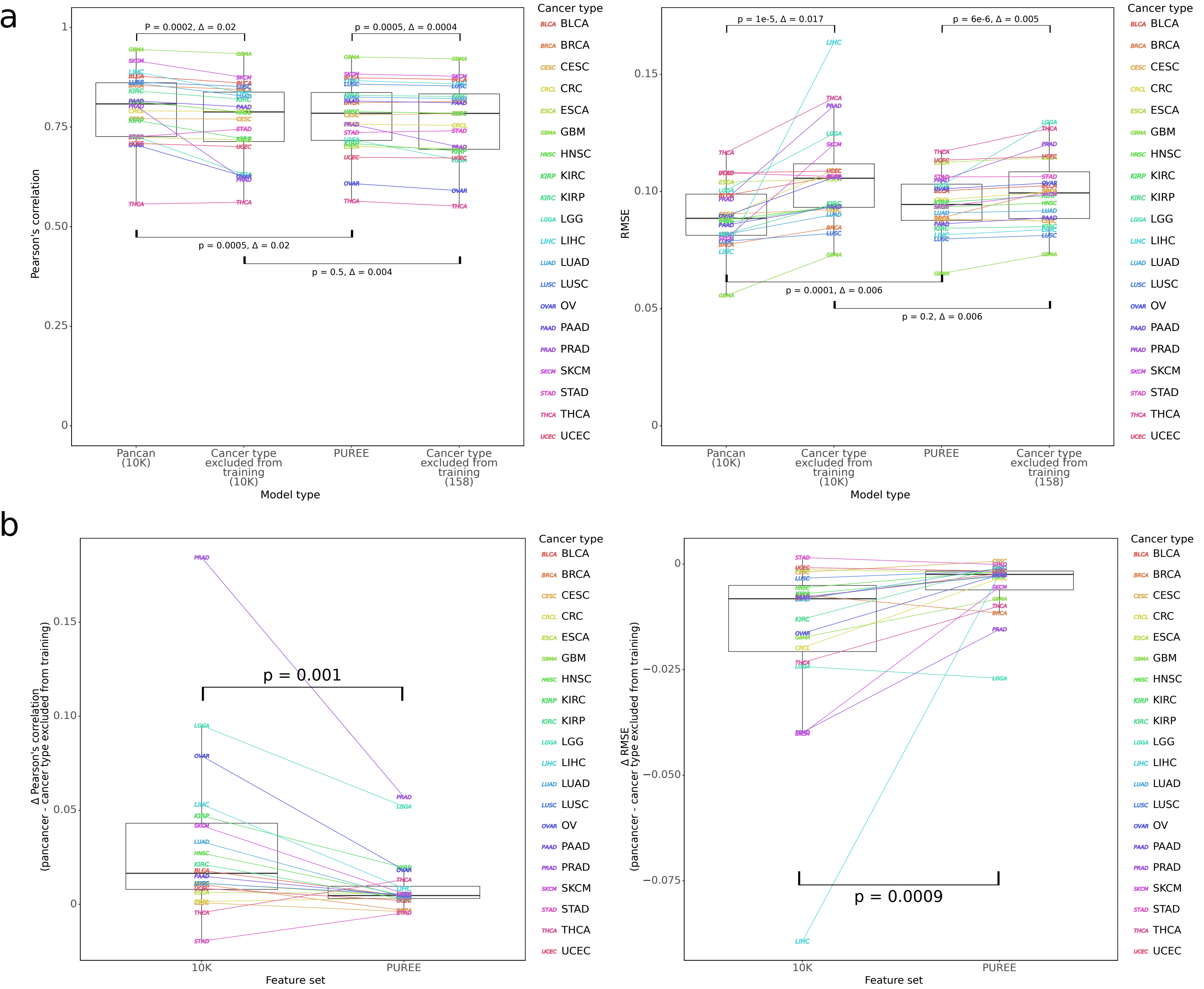

Suppl. Figure 7: One cancer type excluded from training test. a) Means of Pearson's correlation (left) and RMSE (right) for 20 cancer types shown for one cancer type excluded-test for the model built on 10K features versus PUREE. **b)** Differences of means of Pearson's correlation (left) and RMSE (right) for 20 cancer types per feature set (10K versus PUREE), demonstrating the effect of using the reduced feature set. In the boxplots, the lower and upper hinges correspond to the first and third quartiles, the upper whisker extends to the largest value no further than 1.5 of inter-quartile range from the hinge, the lower whisker extends to the smallest value no further than 1.5 of inter-quartile range from the hinge, and points beyond the end of the whiskers are plotted individually. P-values calculated using the Wilcoxon signed-rank test (two-tailed). Delta provided is the difference between medians of metrics across all cancer types.

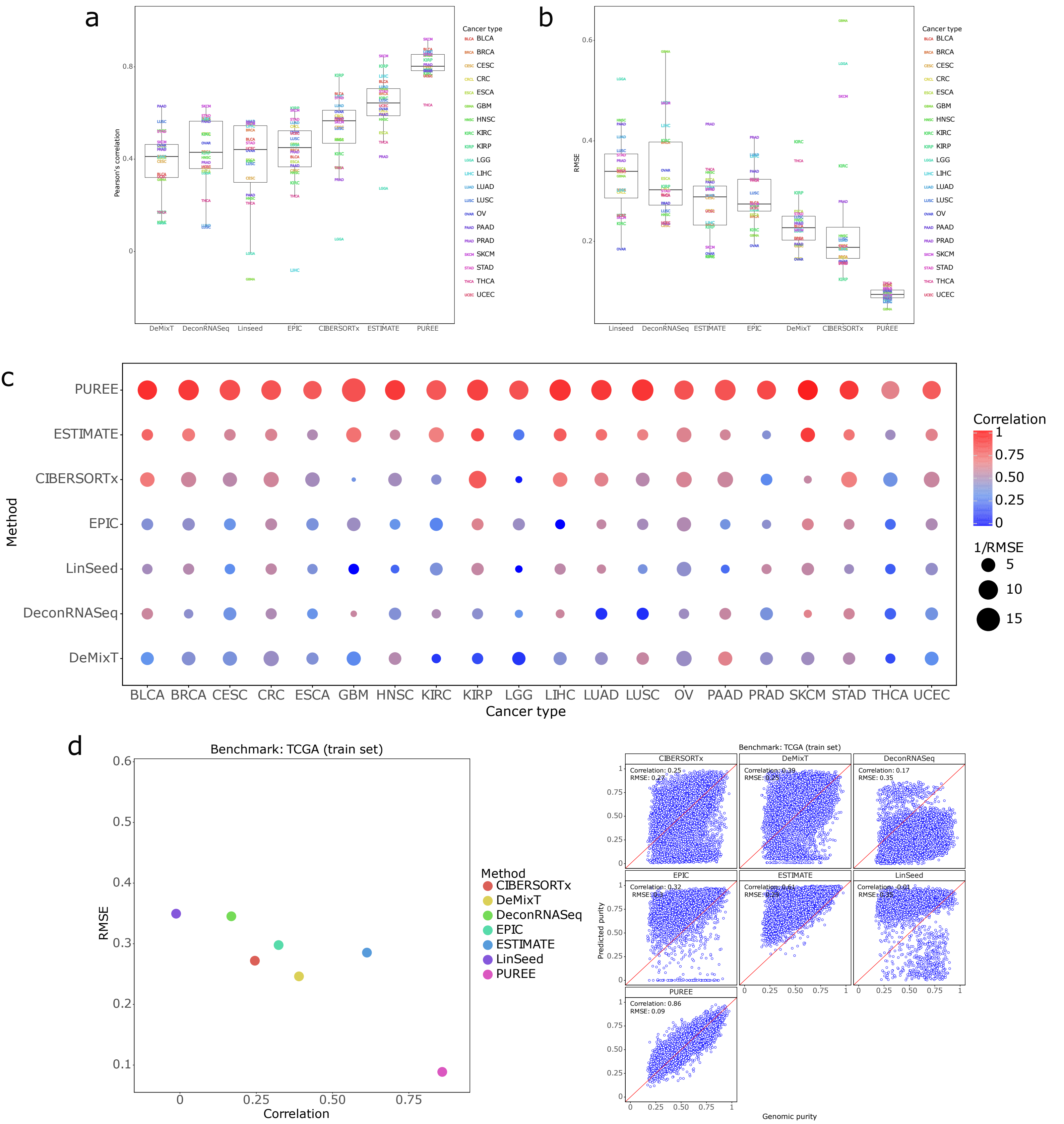

Suppl. Figure 8: RMSE and correlation on TCGA train set. a,b,c) PUREE's and 6 other transcriptomics-based deconvolution methods' performance on TCGA train set (6291 samples), shown across 20 cancer types; mean Pearson's correlation (a) and RMSE (b) separately, and aggregated (c). **d)** Raw data of purity predictions for TCGA train set: aggregated correlation-RMSE plot and raw data. In the boxplots, the lower and upper hinges correspond to the first and third quartiles, the upper whisker extends to the largest value no further than 1.5 of inter-quartile range from the hinge, the lower whisker extends to the smallest value no further than 1.5 of inter-quartile range from the hinge, and points beyond the end of the whiskers are plotted individually. The red diagonal lines are drawn from (0,0) to (1,1), indicating the ideal prediction case.

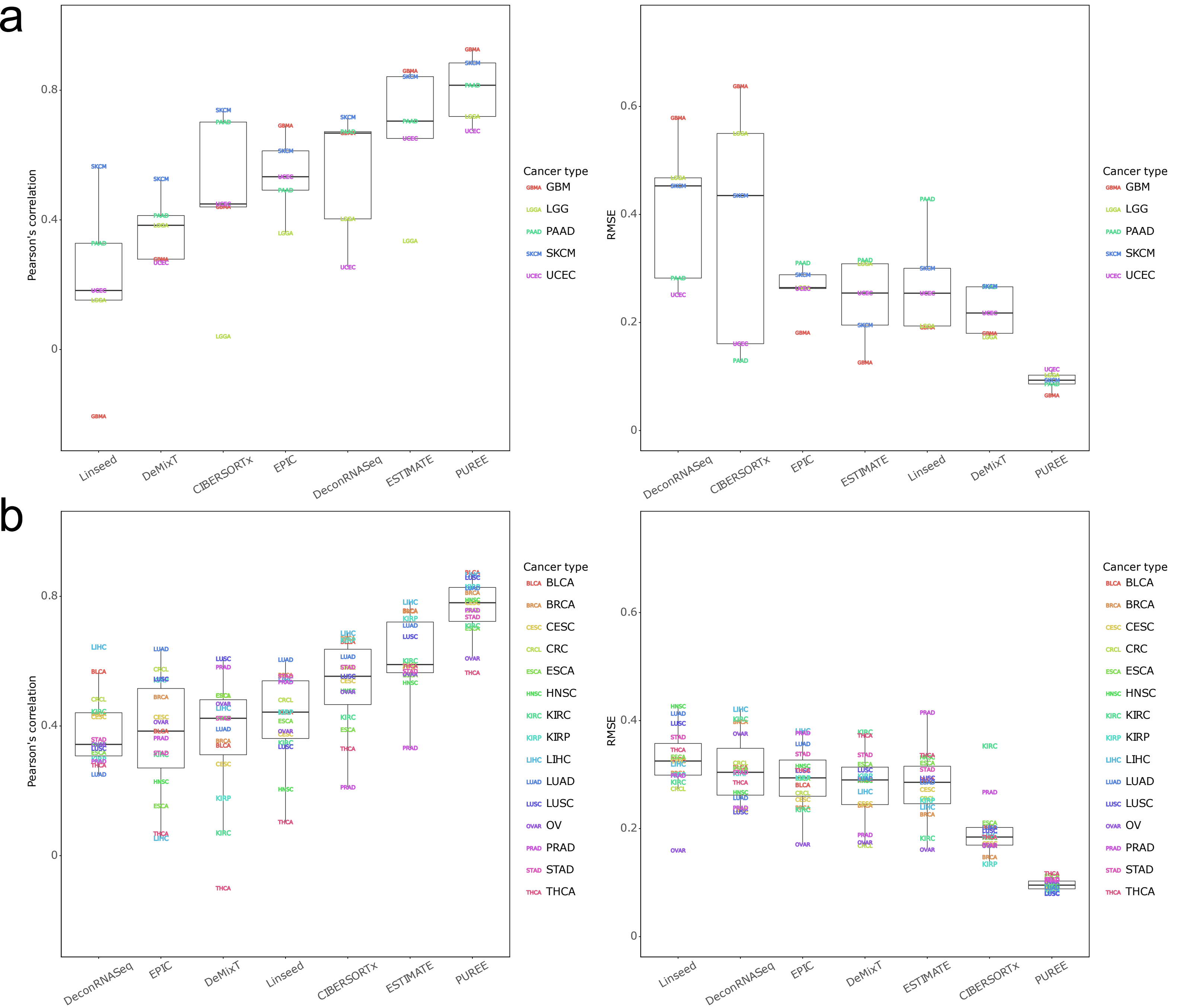

Suppl. Figure 9: Performance of PUREE and 6 other transcriptomics-based methods on TCGA test set for selected cancer types. Mean Pearson's correlation and RMSE of methods per cancer type when compared with genomic tumor purity estimates on the TCGA test data split (1573 samples); **a)** data shown for cancer types with potentially high levels of dissimilar stroma (GBM, LGG, PRAD, SKCM, UCEC), **b)** data shown for other 15 cancer types. In the boxplots, the lower and upper hinges correspond to the first and third quartiles, the upper whisker extends to the largest value no further than 1.5 of inter-quartile range from the hinge, the lower whisker extends to the smallest value no further than 1.5 of inter-quartile range from the hinge, and points beyond the end of the whiskers are plotted individually.

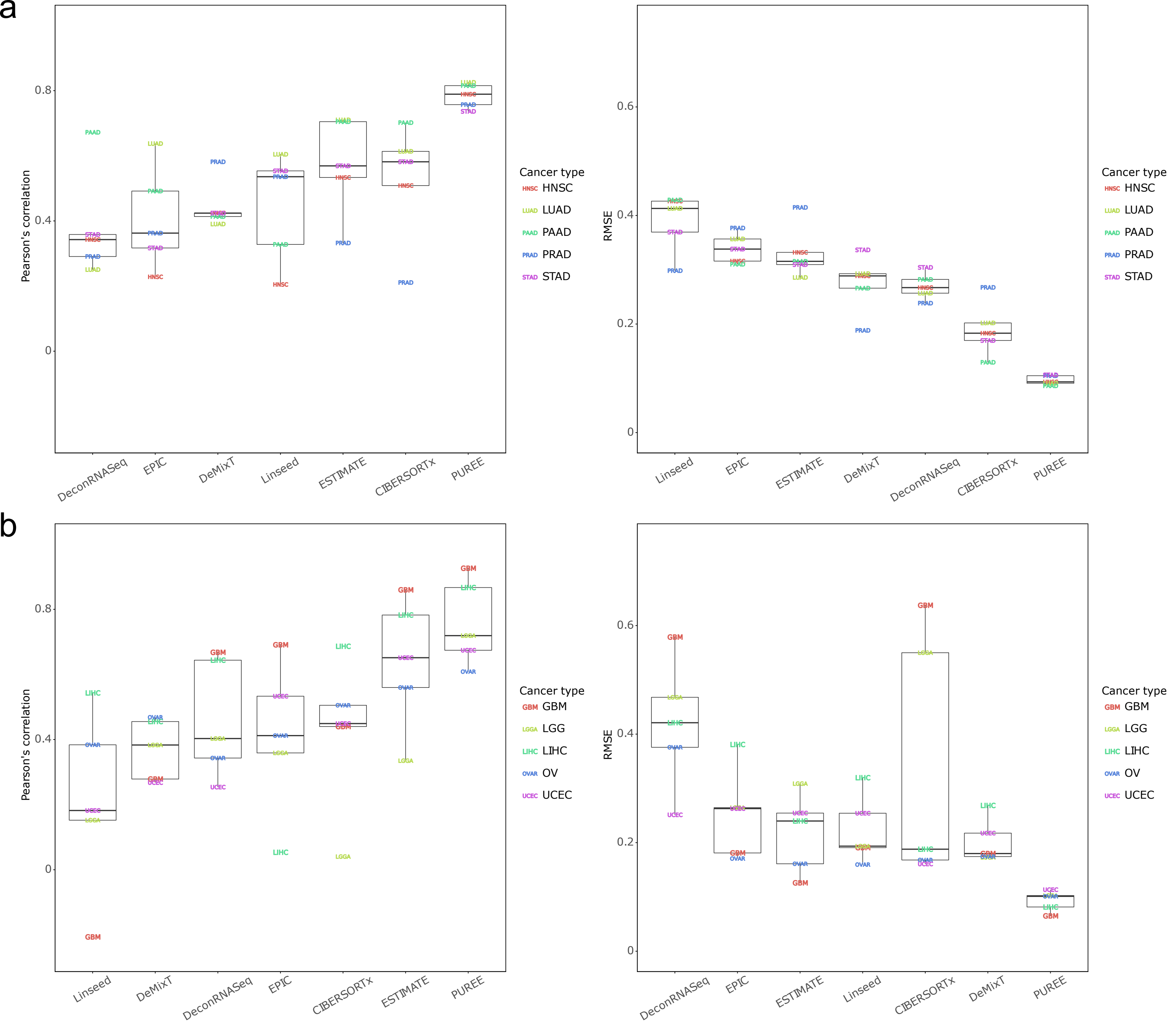

Suppl. Figure 10: Performance of PUREE and 6 other transcriptomics-based methods on TCGA test set for selected cancer types with different median purities. Mean Pearson's correlation and RMSE of methods per cancer type when compared with genomic tumor purity estimates on the TCGA test data split (1573 samples); **a)** cancer types with the lowest median consensus tumor purity (HNSC, LUAD, PAAD, PRAD, STAD), **b)** cancer types with the highest median consensus tumor purity (GBM, LGG, LIHC, OV, UCEC). In the boxplots, the lower and upper hinges correspond to the first and third quartiles, the upper whisker extends to the largest value no further than 1.5 of inter-quartile range from the hinge, the lower whisker extends to the smallest value no further than 1.5 of inter-quartile range from the hinge, and points beyond the end of the whiskers are plotted individually.

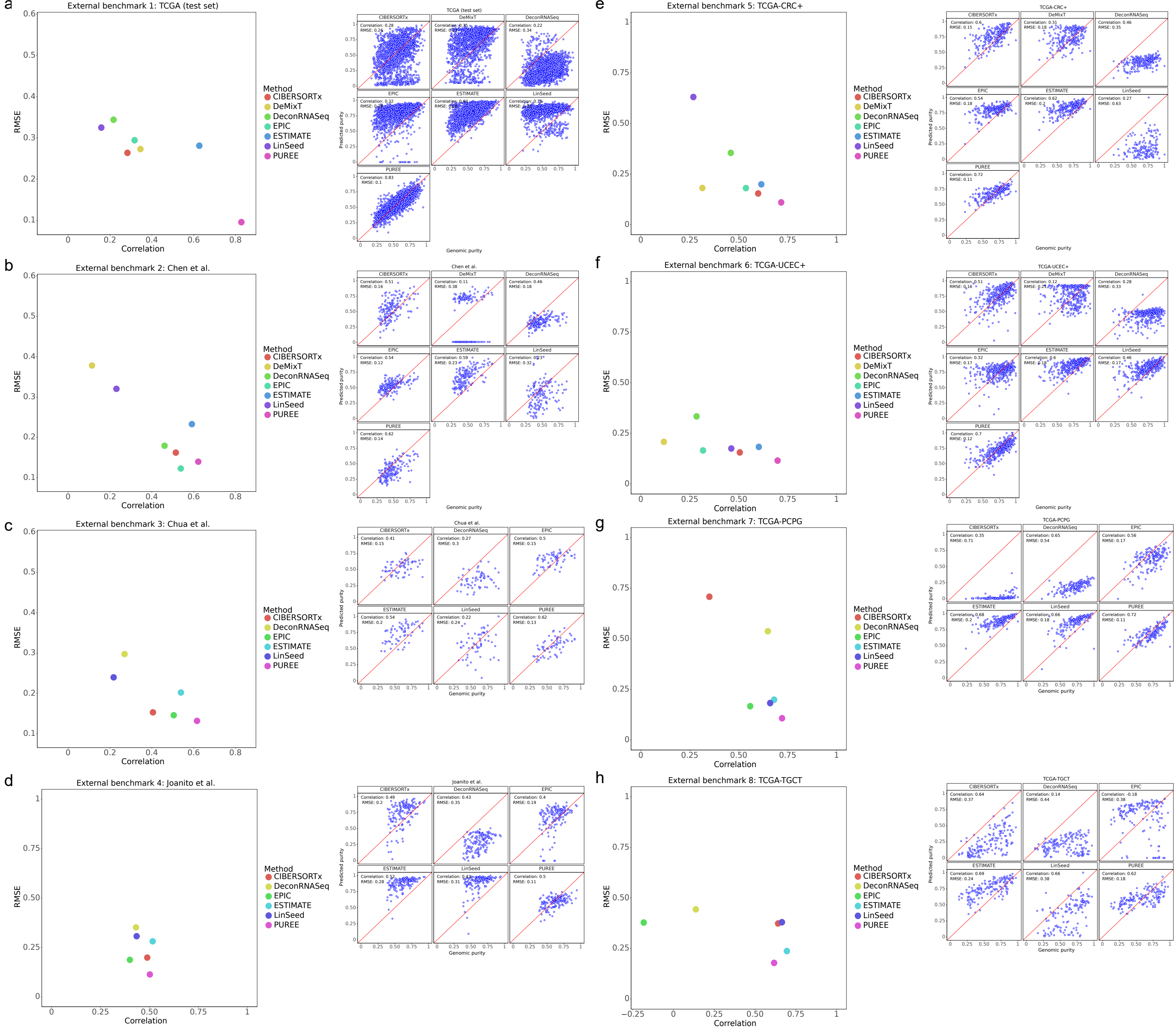

Suppl. Figure 11: External benchmarks' for PUREE's and 6 other transcriptomics-based methods for tumor purity prediction. Aggregated correlation-RMSE performance plots (left) and raw data (right) for **a)** the test portion of TCGA (1573 samples); **b)** Chen et al. cohort, lung cancer (172 samples); **c)** Chua et al. cohort, lung cancer (64 samples); **d)** Joanito et al. cohort, colorectal cancer (153 samples); **e)** TCGA-CRC+ (243 samples), colorectal cancers; **f)** TCGA-UCEC+ (353 samples), uterine endometrial cancer; **g)** TCGA-PCPG, pheochromocytoma and paraganglioma (164 samples) and **h)** TCGA-TGCT (155 samples), testicular cancer. Pearson's r correlation coefficient shown. The red diagonal lines are drawn from (0,0) to (1,1), indicating the ideal prediction case.

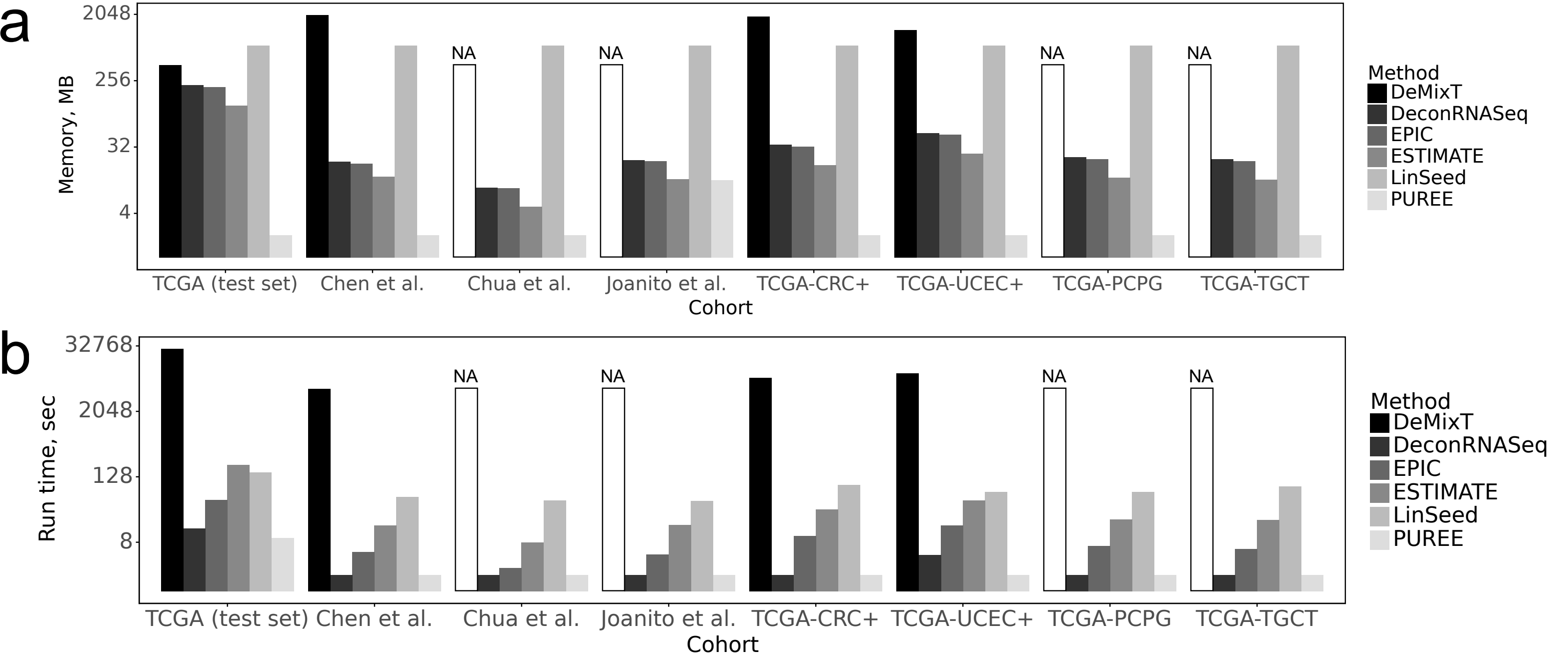

Suppl. Figure 12: Time-memory benchmark on TCGA test split and 7 external cohorts. a) Peak memory used by the prediction function. **b)** Execution time of the prediction function. The time and memory to load the data and save the results were not taken into account. DeMixT could not be run on the Chua et al., Joanito et al., PCPG and TGCT cohorts due to the absence of normal samples there.

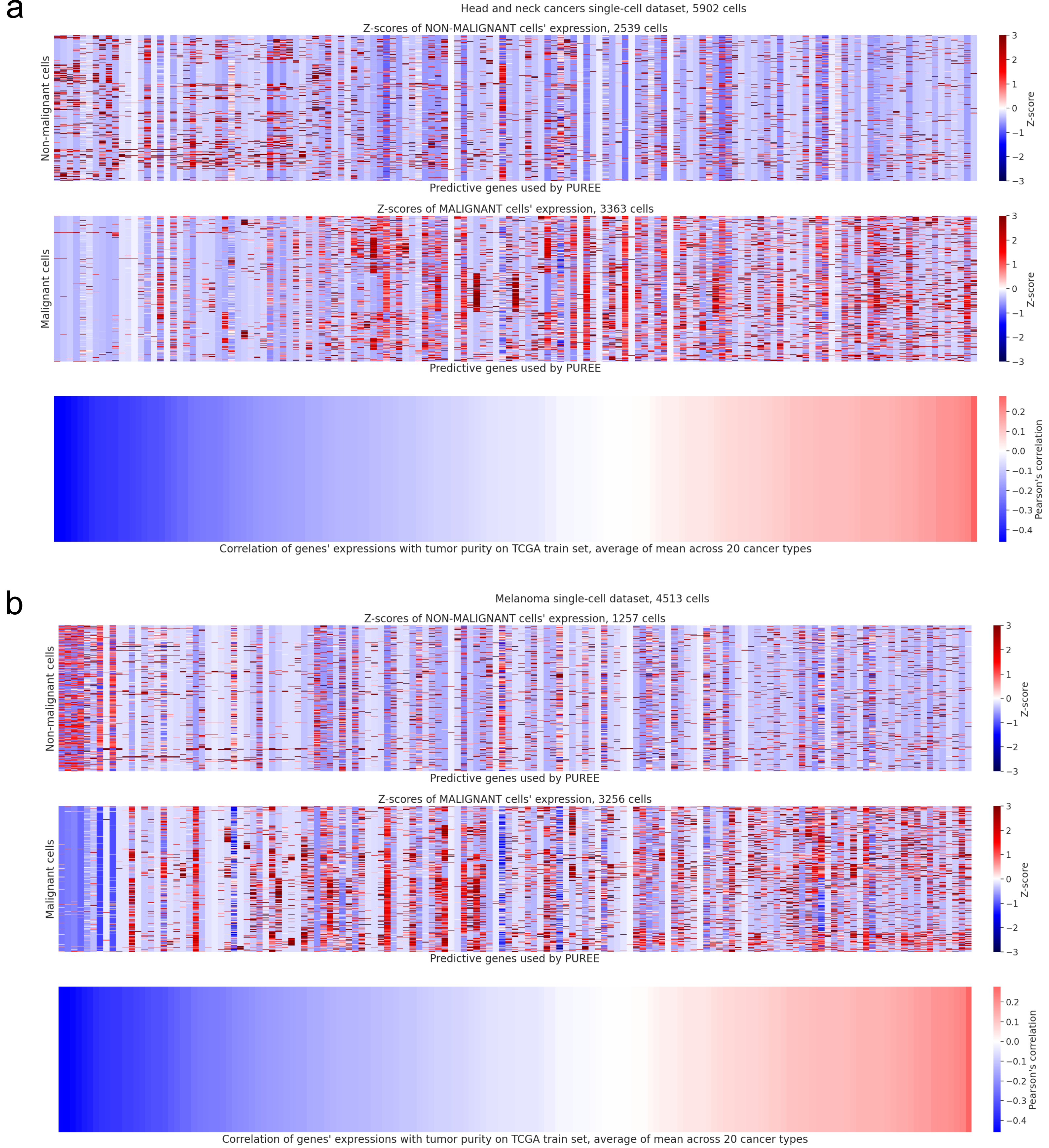

Suppl. Figure 13: Heatmaps of PUREE's genes' expressions in single-cell RNA-seq data. a) Puram et al., 2017, head and neck cancers; **b)** Tirosh et al., 2016, melanoma. Genes (columns) sorted by mean correlations of their expressions with genomic-based tumor purity on TCGA train set. Correlations were computed as means for each cancer type first and then averaged across all cancer types. Genes present in the 158 features of PUREE but missing from the single cell data were dropped, which resulted in 143 genes left in both datasets.

Supplementary References

1. Avila Cobos, F., Vandesompele, J., Mestdagh, P. & De Preter, K. Computational deconvolution of transcriptomics data from mixed cell populations. *Bioinformatics* **34**, 1969–1979 (2018).
2. Newman, A. M. *et al.* Determining cell type abundance and expression from bulk tissues with digital cytometry. *Nat. Biotechnol.* **37**, 773–782 (2019).
3. Racle, J. & Gfeller, D. EPIC: A tool to estimate the proportions of different cell types from bulk gene expression data. in *Methods in Molecular Biology* vol. 2120 233–248 (Humana Press Inc., 2020).
4. Yoshihara, K. *et al.* Inferring tumour purity and stromal and immune cell admixture from expression data. *Nat. Commun.* **4**, 2612 (2013).
5. Barbie, D. A. *et al.* Systematic RNA interference reveals that oncogenic KRAS-driven cancers require TBK1. *Nature* **462**, 108–112 (2009).
6. Carter, S. L. *et al.* Absolute quantification of somatic DNA alterations in human cancer. *Nat. Biotechnol.* **30**, 413–421 (2012).
7. Wang, Z. *et al.* Transcriptome Deconvolution of Heterogeneous Tumor Samples with Immune Infiltration. *iScience* **9**, 451–460 (2018).
8. Zaitsev, K., Bambouskova, M., Swain, A. & Artyomov, M. N. Complete deconvolution of cellular mixtures based on linearity of transcriptional signatures. *Nat. Commun.* **10**, 1–16 (2019).
9. Gong, T. & Szustakowski, J. D. DeconRNASeq: a statistical framework for deconvolution of heterogeneous tissue samples based on mRNA-Seq data. *Bioinformatics* **29**, 1083–1085 (2013).
10. Li, Y. *et al.* Putative biomarkers for predicting tumor sample purity based on gene expression data. *BMC Genomics* **20**, 1021 (2019).
11. Koo, B. & Rhee, J.-K. Prediction of tumor purity from gene expression data using machine learning. *Brief. Bioinform.* **22**, bbab163 (2021).
12. Aran, D., Sirota, M. & Butte, A. J. Systematic pan-cancer analysis of tumour purity. *Nat. Commun.* **6**, 8971 (2015).
13. Wang, L. *et al.* A reference profile-free deconvolution method to infer cancer cell-intrinsic subtypes and tumor-type-specific stromal profiles. *Genome Med.* **12**, 24 (2020).
