# Supplementary material for: PUREE: accurate pan-cancer tumor purity estimation from gene expression data
Source: Commun Biol. 2023 Apr 11;6:394. doi: 10.1038/s42003-023-04764-8 (PMC10090153; doi:10.1038/s42003-023-04764-8)
Supplement: Supplementary file 3 — Reporting Summary [file 42003_2023_4764_MOESM3_ESM.pdf]

nature portfolio

Corresponding author(s): Anders Jacobsen Skanderup

Last updated by author(s): Mar 15, 2023

Reporting Summary

Nature Portfolio wishes to improve the reproducibility of the work that we publish. This form provides structure for consistency and transparency in reporting. For further information on Nature Portfolio policies, see our Editorial Policies and the Editorial Policy Checklist.

Statistics

For all statistical analyses, confirm that the following items are present in the figure legend, table legend, main text, or Methods section.

n/a	Confirmed	
☐	☒	The exact sample size (*n*) for each experimental group/condition, given as a discrete number and unit of measurement
☒	☐	A statement on whether measurements were taken from distinct samples or whether the same sample was measured repeatedly
☐	☒	The statistical test(s) used AND whether they are one- or two-sided<br>*Only common tests should be described solely by name; describe more complex techniques in the Methods section.*
☐	☒	A description of all covariates tested
☐	☒	A description of any assumptions or corrections, such as tests of normality and adjustment for multiple comparisons
☐	☒	A full description of the statistical parameters including central tendency (e.g. means) or other basic estimates (e.g. regression coefficient) AND variation (e.g. standard deviation) or associated estimates of uncertainty (e.g. confidence intervals)
☐	☒	For null hypothesis testing, the test statistic (e.g. *F*, *t*, *r*) with confidence intervals, effect sizes, degrees of freedom and *P* value noted<br>*Give P values as exact values whenever suitable.*
☒	☐	For Bayesian analysis, information on the choice of priors and Markov chain Monte Carlo settings
☒	☐	For hierarchical and complex designs, identification of the appropriate level for tests and full reporting of outcomes
☐	☒	Estimates of effect sizes (e.g. Cohen's *d*, Pearson's *r*), indicating how they were calculated

Our web collection on statistics for biologists contains articles on many of the points above.

Software and code

Policy information about availability of computer code

Data collection	No software or code was used to collect data.
Data analysis	The PUREE method is available as a web service (https://puree.genome.sg/) and the respective Python package (https://github.com/skandlab/PUREE). The version of the code for the PUREE package used to generate the data for the publication was deposited to Zenodo (https://doi.org/10.5281/zenodo.7731645). All other software used in the manuscript is described and cited in the main text and the Methods section.

For manuscripts utilizing custom algorithms or software that are central to the research but not yet described in published literature, software must be made available to editors and reviewers. We strongly encourage code deposition in a community repository (e.g. GitHub). See the Nature Portfolio guidelines for submitting code & software for further information.

Data

Policy information about availability of data

All manuscripts must include a data availability statement. This statement should provide the following information, where applicable:

- Accession codes, unique identifiers, or web links for publicly available datasets
- A description of any restrictions on data availability
- For clinical datasets or third party data, please ensure that the statement adheres to our policy

The tumor purities generated with PUREE and the other methods, as well as the other source data for the figures was deposited to Zenodo (https://doi.org/10.5281/zenodo.7731645). The sources for the public datasets used in this study are provided in the Data Availability section in the manuscript.

Human research participants

Policy information about studies involving human research participants and Sex and Gender in Research.

Reporting on sex and gender	N/A
Population characteristics	N/A
Recruitment	N/A
Ethics oversight	N/A

Note that full information on the approval of the study protocol must also be provided in the manuscript.

Field-specific reporting

Please select the one below that is the best fit for your research. If you are not sure, read the appropriate sections before making your selection.

☒ Life sciences ☐ Behavioural & social sciences ☐ Ecological, evolutionary & environmental sciences

For a reference copy of the document with all sections, see nature.com/documents/nr-reporting-summary-flat.pdf

Life sciences study design

All studies must disclose on these points even when the disclosure is negative.

Sample size	TCGA cohort of 20 cancer types includes 7864 samples with 6291 in the train set and 1573 in the test set. Chen et al. lung cancer cohort consists of 172 samples. Chua et al. lung cancer cohort consists of 64 samples. Joanito et al. colorectal cancer cohort consists of 153 samples. TCGA-CRC+ colorectal cancer cohort consists of 243 samples. TCGA-UCEC+ uterine endometrial cancer cohort consists of 353 samples. TCGA-PCPG pheochromocytoma and paraganglioma cohort consists of 164 samples. TCGA-TGCT cohort consists of 155 samples.<br><br>The head and neck single-cell RNA-seq cohort from Puram et al. consists of 5902 cells, 3363 of which are malignant and 2539 non-malignant. The melanoma single-cell dataset from Tirosh et al. consists of 4513 cells, 3256 of which are malignant and 1257 non-malignant (132 cells with unresolved cell type assignment were dropped).
Data exclusions	No data points were excluded
Replication	N/A
Randomization	Cross-fold validation was applied to validate the final models.
Blinding	N/A

Reporting for specific materials, systems and methods

We require information from authors about some types of materials, experimental systems and methods used in many studies. Here, indicate whether each material, system or method listed is relevant to your study. If you are not sure if a list item applies to your research, read the appropriate section before selecting a response.

Materials & experimental systems

n/a	Involved in the study
☒	☐ Antibodies
☒	☐ Eukaryotic cell lines
☒	☐ Palaeontology and archaeology
☒	☐ Animals and other organisms
☒	☐ Clinical data
☒	☐ Dual use research of concern

Methods

n/a	Involved in the study
☒	☐ ChIP-seq
☒	☐ Flow cytometry
☒	☐ MRI-based neuroimaging
